# Nucleolin regulates the proliferation of vascular smooth muscle cells in atherosclerotic via Aurora B

**DOI:** 10.1111/jcmm.16125

**Published:** 2020-11-20

**Authors:** Hui Sun, Lingjin Huang, Pengfei Liang, Yuting Tang, Cheng Chen, Huan Chen, Xiaofang Lin, Zhengyang Luo, Ying Li, Bimei Jiang, Xianzhong Xiao

**Affiliations:** ^1^ Department of Pathophysiology Sepsis Translational Medicine Key Lab of Hunan Province Xiangya School of Medicine Central South University Hunan China; ^2^ Department of pathophysiology Institute of Cardiovascular Disease and Key Lab for Arteriosclerology of Hunan Province University of South China Hunan China; ^3^ Department of Cardiothoracic Surgery Xiangya Hospital Central South University Hunan China; ^4^ Department of Burns and Plastic Surgery Xiangya Hospital Central South University Hunan China; ^5^ Department of Clinical Laboratory the Second Xiangya Hospital Central South University Hunan China

**Keywords:** atherosclerosis, Aurora B, cell cycle, nucleolin, vascular smooth muscle cells

## Abstract

Vascular smooth muscle cells (VSMCs) play a significant role in atherosclerosis. As a multifunctional protein, nucleolin (NCL) is involved in many important physiological and pathological processes. In this study, we aimed to investigate the role of nucleolin in VSMCs proliferation and cell cycle. The expression of nucleolin increased in VSMCs of mice with aortas advanced plaques. With the left common carotid‐artery ligation‐injury model, immunofluorescence staining revealed that nucleolin and Ki67 expression increased in VSMCs in mice left carotid artery compared with right carotid artery after surgery. POVPC or ox‐LDL up‐regulated nucleolin mRNA and protein expression in a dose‐ and time‐dependent manner in HAVSMCs. POVPC (5μg/ml) or ox‐LDL (50μg/ml) promoted the proliferation of HAVSMCs. Nucleolin ablation relieved the pro‐proliferation role of VSMCs. The cell cycle assay and cell ability results showing that POVPC or ox‐LDL increased the proliferation, but nucleolin ablation inhibited the proliferation of HAVSMCs. And nucleolin ablation can prevent DNA replication at S phase and induce cell cycle arrest in S phase. The bioinformatics database predicts protein‐protein interactions with nucleolin and aurora B. Nucleolin overexpression and ablation affected the expression of aurora B. These findings indicate for the first time that nucleolin actively involved the proliferation of VSMCs via aurora B.

## 1 INTRODUCTION

Atherosclerosis, a chronic inflammatory arterial disease, is associated with dyslipidemia or dyslipoproteinemia and changes in the structural of the blood vessel wall.^1^ Vascular smooth muscle cells (VSMCs) play indispensable roles during the development of atherosclerosis, such as proliferation, migration, apoptosis and autophagy. Under atherosclerosis conditions, VSMC becomes increasingly proliferative and re‐enters the cell cycle.^2^

Nucleolin, also known as NCL or C23, is one of the widely expressed proteins in the nucleolus, cytoplasm, nucleoplasm and cell membrane. As an abundant protein, nucleolin had three main structural domains, which major functions are interaction with different proteins and RNA sequences, cell proliferation, apoptosis, stress response and microRNA processing.^3^, ^4^, ^5^ Latest research found that nucleolin plays mixed roles in tumour growth, virus infection and angiogenesis3. So that nucleolin also a possible target in the development of anti‐tumour and anti‐viral strategies.^6^, ^7^ However, the potential role of nucleolin in VSMCs proliferation and cell cycle is unclear.

Aurora B kinase, a member of the aurora kinase family, which is highly expressed in many cancers, also plays a crucial role in the mitosis.^8^ Aurora B is dynamically distributed during mitosis that the peak of expression level is located in the G2‐M phase.^9^ The nuclear protein Ki67 is expressed in all phases of the cell cycle, but its expression level is strongly up‐regulated in the G1 phase, S phase and G2 phase.^10^ Ki67 is widely used in pathological as a cell proliferation markers. Aurora B is a member of the Aurora kinases family, as an important kinase to adjust the cell normal mitosis, Studies have found that it is Involved in the normal proliferation of vascular smooth muscle cells.^11^

In this study, we aim to investigate the relationship between nucleolin and Aurora B in atherosclerosis plaque and cell lines and elucidate the potential molecular mechanisms of atherosclerotic smooth muscle cells and ameliorate the therapeutic strategies for the treatment of atherosclerosis.

## 2 MATERIALS AND METHODS

### 2.1 Animals and treatment

Male ApoE−/‐ mice on a C57BL/6 background were procured from the Animal Center of Central South University (Changsha, China). All animal experiments were conducted in accordance with the Guidelines of Animal Experimentation, Medical Ethics Committee of Xiangya Hospital, Central South University (No.201402027).

Twenty 6‐ to 8‐week‐old Apo E‐/‐ mice were haphazardly divided into two groups: the control group and the HFD group. The HFD group mice (n = 10 per group) were fed for 12 weeks on a high‐fat diet (HFD), which containing 21% fat and 0.15% cholesterol, the control group mice were fed a normal diet.

Eight‐week‐old C57BL/6 mice (n = 10) underwent surgical operation to induce carotid artery plaque formation, with a collar placed around the left carotid artery and the right carotid as control. After the surgical operation, mice were fed a high‐fat diet and killed 12 weeks later. Then take samples of the left and right carotid arteries.

The mice were killed with isoflurane at the end of the experiment. The aorta and carotid artery were immobilized in 4% paraformaldehyde, and paraffin sections were prepared for haematoxylin‐eosin (HE) staining, immunohistochemical analysis and Immunofluorescence analysis.

### 2.2 Cell culture

The human aortic smooth muscle cells were obtained from the Shanghai Cell Bank of the Chinese Academy of Sciences. The VSMCs were grown in Dulbecco's Modified Eagle Medium (DMEM), which containing 10% foetal bovine serum (FBS, Gibco) and 1% antibiotics (penicillin and streptomy). Ox‐LDL (Oxidized low‐density lipoprotein, Yiyuan biotechnology) or POVPC (1‐palmitoyl‐2‐oxovaleroyl‐sn‐glycero‐3‐phosphorylcholine, Cayman Chemical) was used for treatment of VSMCs, different concentrations of ox‐LDL or POVPC were added into serum medium (10% FBS and 90%DMEM) 24h before experiment. Before treatment with ox‐LDL and POVPC, we will give serum‐free cultured cells to make the cells in the G0 phase and then add drugs for the subsequent experiments. The vascular smooth cells of the third to fifth passages were used for experiments.

### 2.3 Cell transfection experiments

After the vascular smooth muscle cells reached 70%‐80% confluence, that were transfected with pcDNA3.1‐NCL plasmid, at 4μg DNA per 12μL lipofectamine (Megatran 1.0, OriGene Technologies). The pcDNA3.1‐NCL plasmid from Professor Michael B. Kastan (St. Jude Children's Research Hospitaland includes full‐length human nucleolin cDNA. SiRNA‐nucleolin (RIBOBIO) was also transfected using RiboFECT CP Trabsfection reagent (RIBOBIO).

### 2.4 RNA isolation and quantitative Real‐Time PCR analysis

Total RNA was isolated from vascular smooth muscle cells using TRIzol reagent (Takara) following the producer's protocol. Reverse transcription reaction was performed on the total RNA(1μg) by the Prime Script™ RT Reagent Kit (Takara). Measure the nucleolin expression levels using 7500 Fast Real‐Time PCR system (Applied Biosystems). The relative quantity of the target gene mRNA expression was calculated using the formula 2^−△△Ct^. The sequence of the primers was as follows: nucleolin (NM_005381.3) forward primer 5′‐GCACTTGGAGTGGTGAATCAAA‐3’, nucleolin (NM_005381.3) reversed primer 5’‐AAATGCATACCCTTTAGATTTGCC‐3’; β‐actin(NM_001101.5) forward primer 5′‐ GAGCTACGAGCTGCCTGACG‐3’, β‐actin(NM_001101.5) reversed primer 5′‐ GTAGTTTCGTGGATGCCACAG‐3’.

### 2.5 Western blotting, and cell viability

Western blotting and cell viability measurements were performed as previously specified.^12^, ^13^ Anti‐Nucleolin rabbit antibody was obtained from Sigma (1:1000, N2661), anti‐Aurora B rabbit antibody from Cell Signaling Technology (1:1000, 3094T) and anti‐β‐Actin mouse antibody also from Sigma (1:1000, A2228). Anti‐rabbit secondary antibody (1:10 000 dilution, BA1055, Boster) to detect nucleolin protein and aurora B protein and anti‐mouse secondary antibody (1:2500 dilution, BA1050, Boster) to detect β‐Actin protein.

### 2.6 Cell cycle analysis

HAVSMCs were cultured in DMEM (Thermo Fisher Scientific) containing 10% FBS overnight and then were treated with ox‐LDL or POVPC. HAVSMCs were exposed to 25, 50 or 100 µg/mL concentrations of ox‐LDL or were exposed to 2.5, 5.0, 10 or 20 µg/mL concentrations of POVPC. And according to the nucleolin protein expression, we choose the 50 µg/mL concentrations of ox‐LDL and 5 µg/mL concentrations of POVPC for the next experiment. Cell suspensions corresponding to 2 × 10^5^ to 1 × 10^6^ cells were collected. The cells were pelleted by centrifugation. Fixed with 70% ethanol, and propidium iodide (Thermo Fisher Scientific) staining for the cell cycle analysed by flow cytometry. All tests were repeated at least 3 times.

### 2.7 EdU assay

VSMCs were seeded in a 96‐well plate and were cultured overnight and then treated with ox‐LDL (25, 50 or 100 µg/mL) or POVPC (2.5, 5.0, 10 or 20 µg/mL) for 24h. And then added 50 µM of EdU labelling medium to the cell well, the plate allowed incubation at 37°C for 2 hours. The results were expressed as a percentage of EdU‐positive cells from the entire tested population and were calculated from six random fields in three independent experiments.

### 2.8 Statistical analysis

All data were expressed as mean ± SEM. Significant differences were analysed using the Student's t test or one‐way analysis of variance (ANOVA). A value of *P* < .05 indicated a statistically significant difference.

## 3 RESULTS

### 3.1 Nucleolin is up‐regulated during atherosclerosis development

**Figure 1:**
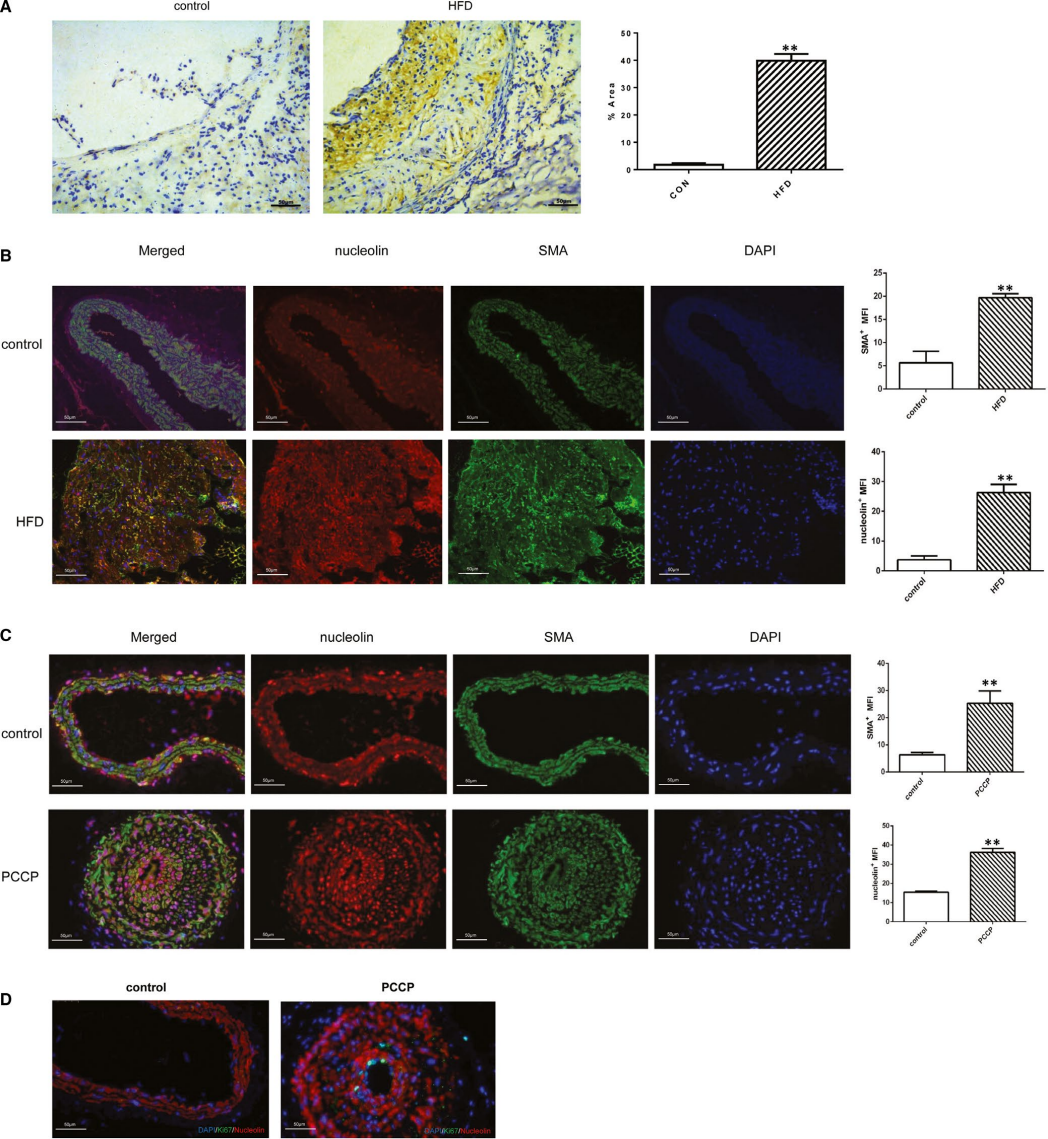
Nucleolin is up‐regulated during atherosclerosis development. (A) Representative immunostaining and quantification for nucleolin on aortic cross sections of ApoE^–/– ^mice fed with chow and high‐fat diets. (Colorimetric images showing nucleolin in brown). Magnification 200×. HFD: high‐fat diet. **, *P* < .01, vs. control group, n = 3. (B) Immunofluorescence analysis showed co‐localization of nucleolin with SMA^+^ cells in ApoE^–/– ^mice fed with chow and high‐fat diets. Immunostaining for nucleolin(red), SMA (green) and DAPI (blue). (C) Immunofluorescence analysis showed co‐localization of nucleolin with SMA^+^ cells in perivascular carotid collarp placement (PCCP) group and conrol group (n = 3). Immunostaining for nucleolin(red), SMA (green) and DAPI (blue). (D) Immunofluorescence analysis showed Ki67 with SMA^+^ cells in perivascular carotid collarp placement (PCCP) group and conrol group (n = 3). Immunostaining for SMA (red), Ki67 (green) and DAPI (blue)

Nucleolin plays a very important role in cell proliferation; however, its specific role in atherosclerosis is largely unknown. Therefore, we first harvested the normal and high‐fat diet feed mice artery and analysed the vascular expression patterns of nucleolin. We observed the expression of nucleolin in the artery by immunohistochemistry. Immunohistochemical assay demonstrated that the protein levels of nucleolin increased in atherosclerosis compared to the sham group (Figure 1A). As shown in Figure 1A, the expression of nucleolin in arterial plaque of mice from HFD group was higher than that in control group (1.825 ± 0.287 vs. 39.86 ± 1.429, *P* < .01, n = 3). By double immunofluorescence staining for nucleolin and SMA, we examined nucleolin expression in the artery. We found that nucleolin was expressed in vascular smooth muscle cells of the thickened intima. Compared with the control group, the expression of nucleolin in the HFD group was significantly increased.

We also checked the result in another model that underwent surgical interventions to induce carotid plaque formation, and a collar around the left carotid artery and the right carotid as control. Furthermore, immunofluorescence staining revealed that nucleolin expression highly increased in VSMCs in the area of intimal thickening and in the medial layer in mice left carotid artery (PCCP) compared with right carotid artery after surgery. Moreover, we checked Ki67 expression by immunohistochemistry in the control group and in the PCCP group. Immunohistochemical assay demonstrated that the protein levels of Ki67 were increased in PCCP group compared with sham group. These data suggest that nucleolin plays a key role in vascular smooth muscle cells of atherosclerosis.

### 3.2 POVPC or ox‐LDL up‐regulated the mRNA and protein expression of nucleolin in HAVSMCs

**Figure 2:**
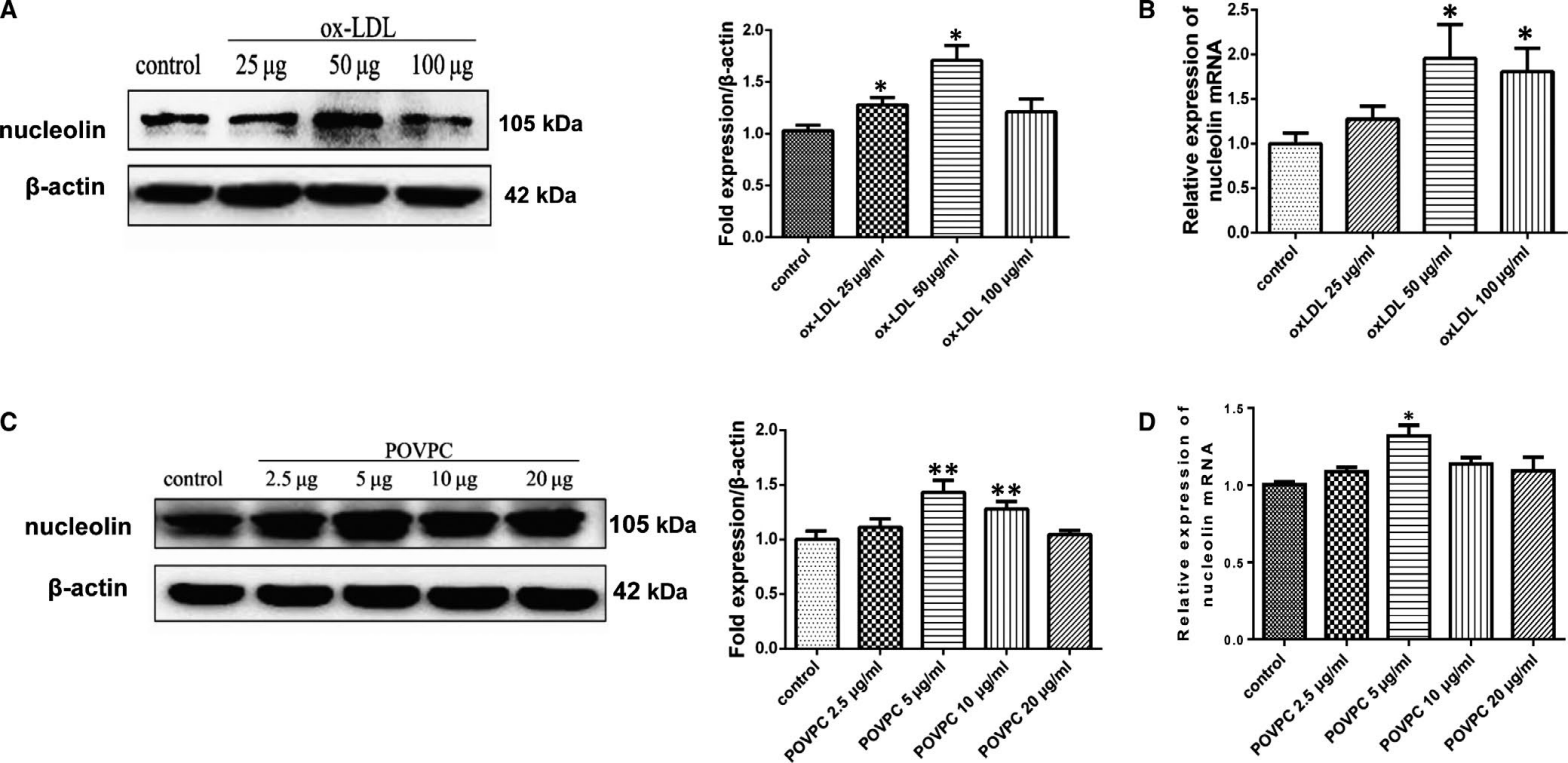
POVPC or ox‐LDL up‐regulated nucleolin mRNA and protein expression in HAVSMCs. (A and C) Representative Western blot showing nucleolin levels in VSMCs treated with POVPC or ox‐LDL. *, *P* < .05, **, *P* < .01, compared with control, n = 3. (B and D) RT‐qPCR gene expression analysis of VSMCs treated with different stimuli. Unpaired 2‐tailed Student's t test was used to compare means for A‐D. *, *P* < .05, compared with control, n = 3

Atherosclerosis is an arterial disease associated with dyslipidemia and changes in the composition of the blood vessel wall. Vascular smooth muscle cells (VSMCs) proliferation plays an important role in the development of atherosclerosis. Therefore, we chose vascular smooth muscle cells as the research object. To confirm these results, human aortic SMCs were stimulated by ox‐LDL or POVPC. After treatment for 24h, nucleolin expression was analysed by quantitative reverse‐transcription PCR (RT‐qPCR) and Western bolting (WB) analysis. Ox‐LDL and POVPC resulted in dramatic up‐regulation of nucleolin expression, displaying dose dependence. Ox‐LDL as a stimulator that can induce VSMC proliferation. HAVSMCs were exposed to 25, 50 or 100 µg/mL concentrations of ox‐LDL. Western blot analysis showed that the expression of nucleolin protein was induced significantly in the 50µg/mL groups, which was 1.711 times higher than that of the control group (*P* < .05) (Figure 2A). Compared with the control group, the mRNA levels of nucleolin increased by 1.936 times in 50µg/mL groups and 1.936 times in 100 µg/mL groups (*P* < .05). HAVSMCs were exposed to 2.5, 5.0, 10 or 20 µg/mL concentrations of POVPC. The Western blot analysis showed that nucleolin protein expression was induced significantly in the by 1.433 times in 5.0 µg/mL groups and 1.278 times in 10µg/mL groups, compared with the control group (*P* < .01) (Figure 2C). The real‐time PCR analysis showed that the nucleolin mRNA levels increased by 1.321 times in 5.0 µg/mL groups, respectively, compared with the control group (*P* < .05). According to the results, we chose the dose 5 μg/mL of POVPC or 50 μg/mL of ox‐LDL in the subsequent experiments. As well, our results showed that ox‐LDL or POVPC treatment increased nucleolin expression in HAVSMCs.

### 3.3 POVPC or Ox‐LDL induced the cell cycle changes in vascular smooth muscle cells

**Figure 3:**
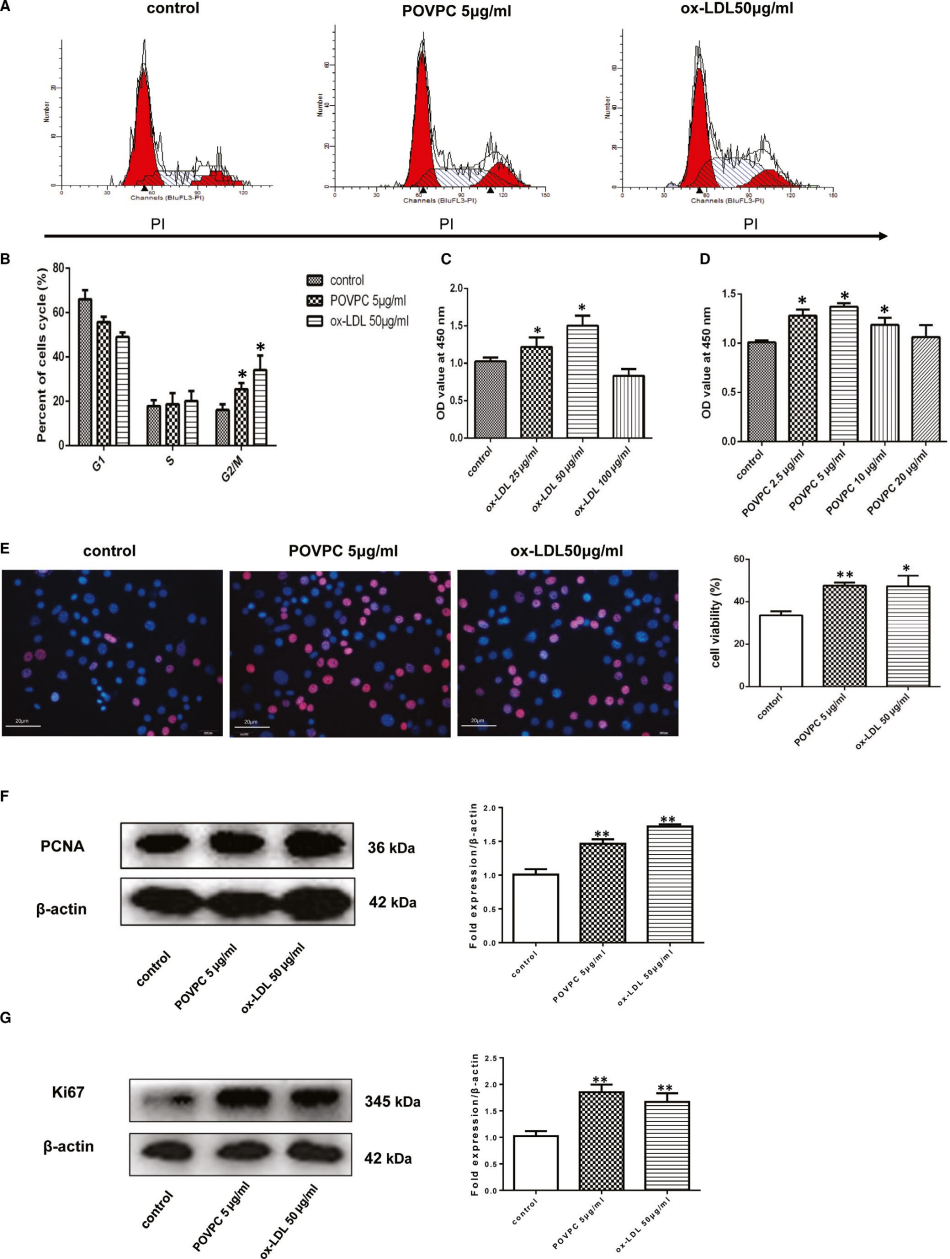
POVPC‐induced or Ox‐LDL‐induced the cell cycle changes in vascular smooth muscle cells. (A). POVPC‐induced or ox‐LDL‐induced the cell cycle changes in vascular smooth muscle cells. The cell cycle distribution was determined by assessing the individual nuclear DNA content reflected by the fluorescence intensity of incorporated propidium iodide. Flow cytometric analysis of VSMCs without any treatment (control), VSMCs induced by POVPC or ox‐LDL, respectively. (B) Percentages of cells in G0/G1, S and G2/M phases after the indicated treatments. Representative flow cytometry graphs are shown, and results are expressed as the mean ± standard deviation from three independent experiments. (C‐D) VSMCs were treated for 24 h with different concentrations of POVPC or ox‐LDL for 24h. Cell viability was assessed by the CCK‐8 method. POVPC or ox‐LDL treatment significantly increased the cell viability. Values are expressed as the mean ± standard deviation from three independent experiments. VSMC, vascular smooth muscle cell; ox‐LDL, oxidized low‐density lipoprotein; POVPC, 1‐palmitoyl‐2‐oxovaleroyl‐sn‐glycero‐3‐phosphorylcholine. n = 10, * *P* < .05, vs. the control group. (E) Representative images of EdU staining. Edu staining was revealed with a red fluorescence signal; bar diagrams showing the number of EdU‐positive cells; Immunostaining for SMA (red) and hoechst (blue), n = 9, *P < .05, ** *P* < .01, vs. the control. (F) Representative Western blot showing PCNA levels in VSMCs treated with POVPC or ox‐LDL. n = 3, ** *P* < .01, vs. the control. (G) Representative Western blot showing Ki67 levels in VSMCs treated with POVPC or ox‐LDL. n = 3, **, *P* < .01, vs. the control group. Unpaired 2‐tailed Student's t test was used to compare means for A‐G

To elucidate the effect of nucleolin on cell cycle regulation, flow cytometry was used to evaluate the cell cycle distribution of ox‐LDL‐induced or POVPC‐induced VSMCs. As shown in Figure 3A‐B, POVPC or ox‐LDL treatment increased the percentage of VSMCs in S and G2/M phases, especially in G2/M phase and correspondingly reduced the percentage of cells in G0/G1 phase. The percentage of cells at G0/G1, S and G2/M phases in the ox‐LDL stimulated group was 52.66 ± 1.90, 26.26 ± 4.12 and 21.09 ± 2.23%, the POVPC stimulated group was 54.36 ± 5.88, 31.92 ± 5.73 and 13.71 ± 3.01%, and the control group was 62.70 ± 1.84, 26.36 ± 0.70 and 10.95 ± 1.23% in Figure 3B. These results showed that nucleolin may improve VSMCs proliferation induced by POVPC or ox‐LDL; these effects may be related to the induction of cell cycle arrest in G0/G1 phase. Treatment of VSMCs with POVPC or ox‐LDL resulted in a dose‐dependent reduction of cell growth as monitored by CCK8 colorimetric cell viability assay. We found that the two groups, as 5.0 µg/mL POVPC group and 50 µg/mL ox‐LDL group have the best cell ability (Figure 3C‐D). The results of the EdU assay showed that POVPC or ox‐LDL treatment increased VSMCs proliferation (Figure 3E). As shown in Figure 3F‐G, the expression of Ki67 and PCNA in vascular smooth muscle cells treated with POVPC or ox‐LDL was higher than that of control group. Our data indicated that POVPC or ox‐LDL treatment significantly increased vascular smooth muscle cell viability.

### 3.4 The cell proliferation ability changed after interference with the expression of nucleolin

**Figure 4:**
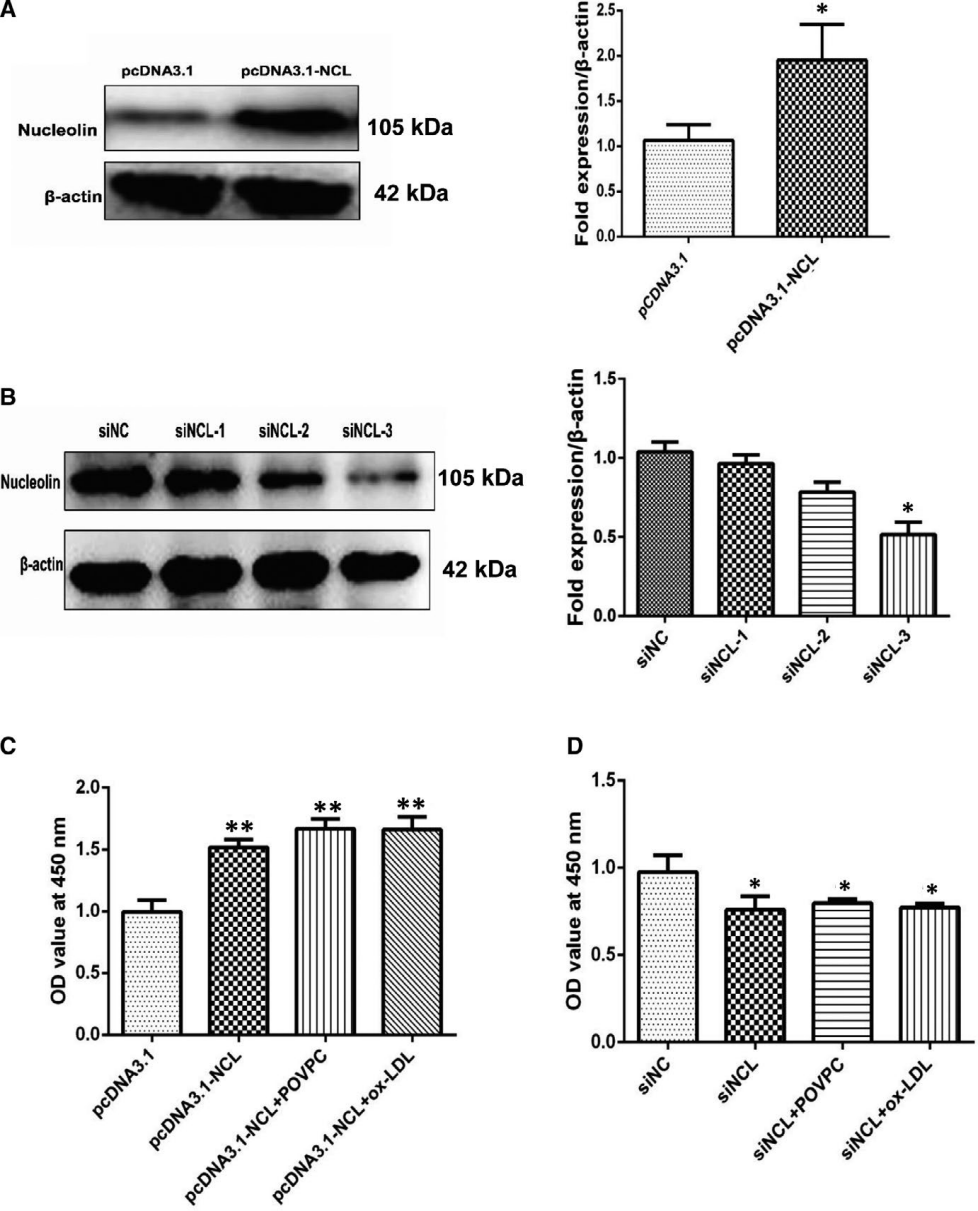
The cell proliferation ability changed after interference with the expression of nucleolin. (A) The protein expression of nucleolin fusion protein in vascular smooth muscle cells after transfection with pcDNA3.1‐NCL for 48 hours. The right‐hand side showed the grey ratio analysis of nucleolin/ β‐actin. *, *P* < .05, compared with vector control (pcDNA3.1), n = 3. (B) The expression of nucleolin protein in vascular smooth muscle cells after transfection with nucleolin specific siRNA. The right‐hand side showed the grey ratio analysis of nucleolin/actin. *, *P* < .05, compared with negative control (siNC), n = 3. (C) The effect of nucleolin siRNA on cell viability in VSMCs. *, *P* < .05, vs siNC group, n = 6. (D) The effects of nucleolin overexpression on cell viability in VSMCs. **, *P* < .01, vs pcDNA3.1 group, n = 6. *P*‐values were determined using the two‐tailed Student's t test for comparing two groups and one‐way ANOVA for comparing multiple groups

Several studies have found that nucleolin played a significant role in cell proliferation, but its role in vascular smooth muscle cells of atherosclerosis remains unclear. Therefore, we transfected the vascular smooth muscle cells with a recombinant vector, which carries nucleolin cDNA (pcDNA3.1‐NCL), and analysed the nucleolin expression. It is shown in Figure 4A that the expression level of pcDNA3.1‐NCL fusion protein in transfected VSMCs cells for 48hr increased, compared with the cells transfected with pcDNA3.1(vector control). We also examined the effect of nucleolin knocked down in vascular smooth muscle cells in parallel. The nucleolin knocked down through its specific siRNA(siNCL) was confirmed by Western blotting as shown in Figure 4B. We observed a significant reduction in its expression (*P* < .01) compared with the cells transfected with control siRNA(siNC).

Then, we used the CCK‐8 assay to measure the proliferation of VSMCs. These data show that nucleolin overexpression obviously increased cell viability (Figure 4C). When knock‐down of nucleolin drastically reduced the proliferation of VSMCs (Figure 4D). The results showed that nucleolin played a vital role in the proliferation of vascular smooth muscle cells.

### 3.5 Nucleolin overexpression or down expression change the cell cycle progression of VSMCs

**Figure 5:**
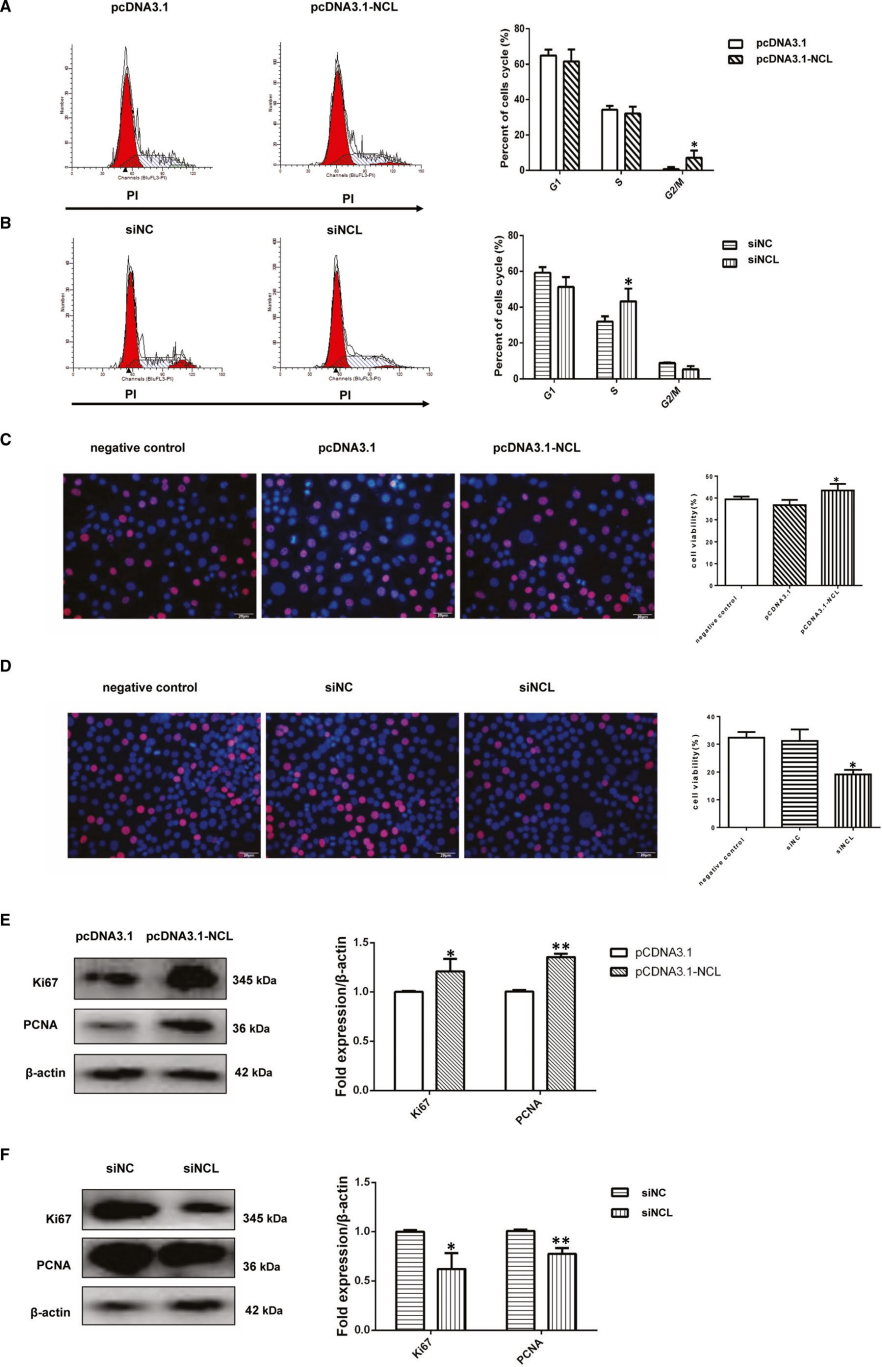
Nucleolin ablation or overexpression change the cell cycle progression of VSMCs. (A) The cell cycle changes in vascular smooth muscle cells after transfected with pcDNA3.1 or pcDNA3.1‐NCL. Percentages of cells in G0/G1, S and G2/M phases after the indicated transfected treatments. Representative flow cytometry graphs are shown, and results are expressed as the mean ± standard deviation from three independent experiments, *, *P* < .05, vs pcDNA3.1 group (vector control), n = 3. (B) The cell cycle changes in vascular smooth muscle cells after transfected with siNCL or siNC. Percentages of cells in G0/G1, S and G2/M phases after the indicated transfected treatments. *, *P* < .05, compared with siNC (negative control), n = 3. (C) Representative images of EdU staining in vascular smooth muscle cells after transfected with negative control, pcDNA3.1 or pcDNA3.1‐NCL. EdU‐positive cells increased significantly in pcDNA3.1‐NCL group and the percentage of EdU‐positive cells was calculated, EdU (red) and hoechst (blue), n = 6, *, *P* < .05, compared with the pcDNA3.1 group. (D) Representative images of EdU staining in vascular smooth muscle cells after transfected with negative control, siNC or siNCL. EdU‐positive cells(red) decreased in siNCL group and the percentage of EdU‐positive cells was calculated, n = 6, *, *P* < .05, compared with the siNC group. (E) Representative Western blot showing PCNA and Ki67 levels in VSMCs after transfected with pcDNA3.1 or pcDNA3.1‐NCL group. n = 3, *, *P* < .05, **, *P* < .01, compared with the pcDNA3.1 group. (F) Representative Western blot showing PCNA and Ki67 levels after transfected with siNC or siNCL group. n = 3, *, *P* < .05, **, *P* < .01, compared with the siNC group. P‐values were determined using the two‐tailed Student's *t* test for comparing two groups and one‐way ANOVA for comparing multiple groups

We investigated the role of the expression of nucleolin protein in the cell cycle of VSMCs. Therefore, we performed the flow cytometry on nucleolin overexpression of vascular smooth muscle cells (pcDNA3.1 group and pcDNA3.1‐NCL group). More cells were in G2/M phases in pcDNA3.1‐NCL group, compared with pcDNA3.1 group (1.237 ± 0.148% vs. 7.133 ± 2.359% at 24h) (Figure 5A). And then, we also tested the flow cytometry on vascular smooth muscle cells with ablation nucleolin expression (siNCL group and siNC group). At the same time point, there were more cells in S phases In siNCL group, compared with NC group (8.777 ± 0.217% vs. 4.697 ± 1.197% at 24h) (Figure 5B). It means that nucleolin ablation can effectively prevent DNA replication at S phase and induce cell cycle arrest in the S phase.

The results of EdU assay showed that ablation nucleolin expression inhibited VSMCs proliferation, and nucleolin overexpression promoted the vascular smooth muscle cells proliferation (Figure 5C‐D). We also analysed the expression of Ki67 and PCNA in VSMCs cells either nucleolin ablation or nucleolin overexpression. VSMCs transfected with pcDNA3.1‐NCL plasmid significantly increased Ki67 and PCNA (Figure 5E). But in siRNA‐nucleolin (siNCL) group exhibited considerably lower levels of Ki67 and PCNA expression in VSMCs cells (Figure 5F).

### 3.6 Aurora B is a direct target of nucleolin in VSMCs

**Figure 6:**
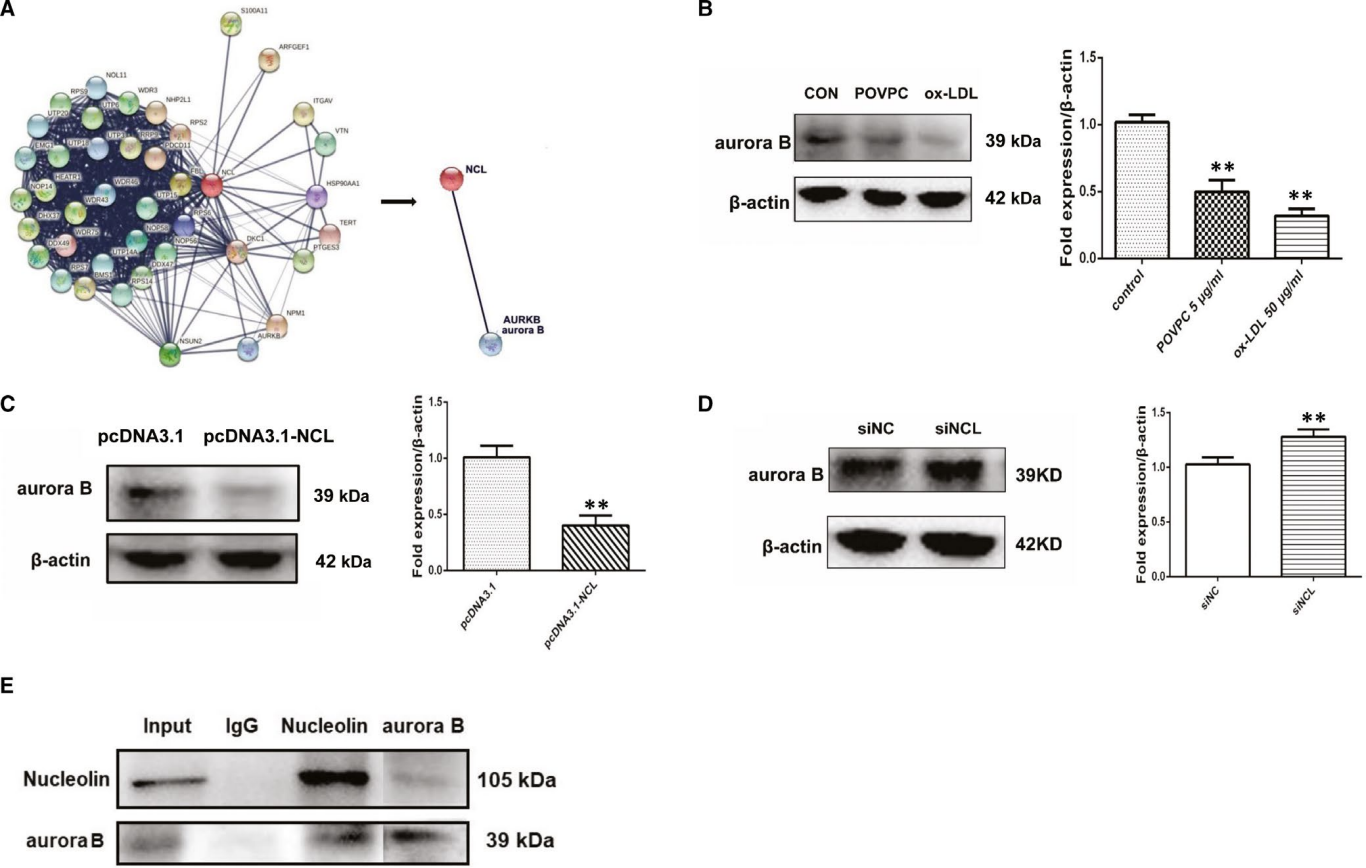
Aurora B is a direct target of nucleolin in VSMCs. (A) Aurora B as a potential target gene by previous studies and String software (https://string‐db.org/). (B) The protein expression of aurora B fusion protein in vascular smooth muscle cells treated with POVPC or ox‐LDL. n = 3; **, *P* < .01 vs control group. (C) VSMCs were transfected with pcDNA3.1 or pcDNA3.1‐NCL. The protein level of aurora B was measured by Western blot. n = 3; **, *P* < .01 vs pcDNA3.1 (vector control). (D) VSMCs were transfected with siNCL or siNC. The protein level of aurora B was measured by Western blot. n = 3; **, *P* < .01 vs siNC (negative control). (E) Interaction of nucleolin and aurora B tested by immunoprecipitation. Lane 1 represented whole cell lysate. Lane2‐4 represented the proteins precipitated by control IgG, anti‐nucleolin, anti‐aurora B. The upper band indicated nucleolin, and the lower band indicated aurora B. Each experiment was repeated three times. P‐values were determined using the two‐tailed Student's t test for comparing two groups and one‐way ANOVA for comparing multiple groups

**Figure 7:**
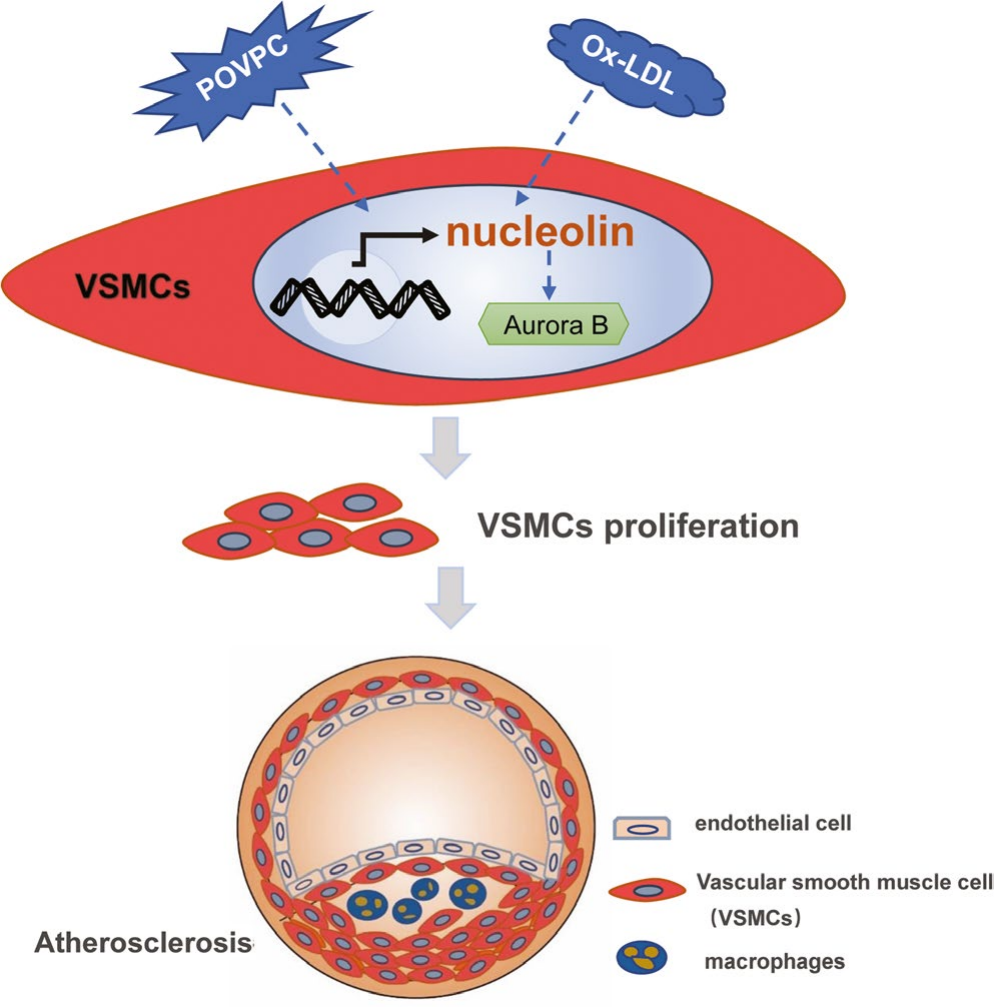
Schematic summary of the findings. POVPC or ox‐LDL up‐regulated nucleolin mRNA and protein expression in HAVSMCs, and then the cell cycle changes. Nucleolin may interact with aurora B to regulate the cell cycle and proliferation of vascular smooth muscle cells

To search the target of nucleolin, we given a demonstration of aurora B as a possible target gene by previous studies and String software(https://string‐db.org/) (Figure 6A). Whether the role of nucleolin against POVPC or ox‐LDL induced vascular smooth muscle cells proliferation involves aurora B is still unclear. Firstly, the protein expression of aurora B in vascular smooth muscle cells treated with POVPC or ox‐LDL was assessed by Western blotting analysis. The expression of aurora B in vascular smooth muscle cells treated with POVPC or ox‐LDL was lower than the control group in Figure 6B (*P* < .05). Furthermore, we also analysed the aurora B expression in VSMCs by nucleolin overexpression or nucleolin ablation. VSMCs transfected with pcDNA3.1‐NCL plasmid significantly reduced aurora B, in comparison with non‐transfected VSMCs or cells transfected with pcDNA3.1(*P* < .01). In parallel, nucleolin ablation by siRNA‐nucleolin (siNCL) exhibited considerably higher levels of aurora B expression in VSMCs (*P* < .01) (Figure 6D). We used the protein immunoprecipitation method to observe the interaction between nucleolin and aurora B in vascular smooth muscle cells under physiological conditions. As shown in Figure 6E, aurora B interacts with nucleolin under physiological conditions. It is suggested that nucleolin may interact with aurora B to regulate the cycle and proliferation of vascular smooth muscle (Figure 7).

## 4 DISCUSSION

Atherosclerosis is a chronic vascular inflammatory disease characterized by atherosclerotic plaques, which are composed of vascular smooth muscle cells, endothelial cells, platelets, leukocytes and other cells. The study found that inhibition of VSMC proliferation is a significant curative strategy for the precaution and treatment of atherosclerosis.^2^, ^14^ In our study, nucleolin regulates the vascular smooth muscle cells proliferation in atherosclerosis by regulating the expression of aurora B.

Nucleolin is the plentiful DNA‐, RNA‐ and protein‐binding protein in the nucleolus, which major functions are cell proliferation, cytokinesis, angiogenesis, apoptosis regulation and among others.^3^, ^4^ However, the potential role of nucleolin in VSMCs proliferation and cell cycle is unclear. Our study showed that nucleolin was increased in vascular smooth muscle cells in the atherosclerosis plaque of apoE^‐/‐^ mice. With the left common carotid‐artery ligation‐injury model, nucleolin and Ki67 expression significantly increased in VSMCs in mice left carotid artery compared with right carotid artery after surgery. These results suggest that nucleolin plays a key role in vascular smooth muscle cells of atherosclerosis.

It is gradually recognized that ox‐LDL plays a vital role in promoting the occurrence, progression and plaque destabilization of atherosclerosis.^15^ Multiple studies have designated that ox‐LDL can stimulate the VSMCs proliferation.^16^, ^17^ Therefore, the proliferation of VSMCs induced by ox‐LDL in the arterial is considered to play a crucial role in the development of atherosclerotic lesions. POVPC (1‐palmitoyl‐2‐(5‐oxovaleroyl)‐sn‐glycero‐3‐phosphocholine) is one of the LDL oxidation products that is found in atherosclerotic lesions is a proinflammatory lipid.^18^ Previous studies have shown that POVPC is involved in the growth and differentiation of vascular smooth muscle cells and participates in cholesterol synthesis. The present study indicates that POVPC is related to vascular proliferation and chronic inflammation.^19^ In our research, POVPC or ox‐LDL up‐regulated nucleolin mRNA and protein expression in a dose ‐dependent manner in HAVSMCs. We noticed High‐dose oxLDL and POVPC were affected vascular smooth cells proliferation, so we choose an appropriate concentration to stimulate cells.

Nucelolin plays key roles in cell proliferation and other cellular processes. These findings showed that POVPC or ox‐LDL induced VSMC proliferation and provided nucleolin is involved in the process of cell proliferation. The cell cycle regulated cell proliferation, which is a highly regulated process and involves a series of complex events.^20^ The cell cycle contains three different phases: G0/G1 phase, S phase and G2/M phase.^21^ Under normal conditions, VSMCs proliferate slowly and remain in the G0/G1 phase. When proliferative stimulation, VSMCs began to proliferate and re‐enter the cell cycle. In our study, flow cytometric analysis showed that the proliferation of VSMCs was promoted by nucleolin and increased the S‐phase and G2/M phase, whereas decreasing in the G0/G1‐phase population. However, nucleolin ablation significantly increased the cells in S phase, and cell cycle repression is in S phase. In present study, we were concerned that ox‐LDL or POVPC up‐regulates the expression of nucleolin in vascular smooth muscle cells. We found that increased expression of nucleolin promoted proliferation and cycle changes of vascular smooth muscle cells. In atherosclerosis, the abnormal proliferation of vascular smooth muscle cells (VSMC) is the basic cytopathological basis for its occurrence and development. Therefore, we believe that nucleolin is pro‐atherogenic in VSMCs of atherosclerosis. We know that the occurrence and development of diseases are regulated by many aspects, and atherosclerosis is not only involved in vascular smooth muscle cells. Moreover, it is not clear whether the expression of nucleolin changes at different stages of atherosclerosis, and its potential role and mechanism in the development of atherosclerosis are still unclear.

CDKs and Cyclins are positive regulators that are directly involved in cell proliferation.^22^ We notice the Aurora kinases B,^8^, ^9^ that is a member of the silk‐threonine kinase family, and its coding gene is located in human chromosome 17p13.1, which possesses a conserved catalytic domain and a N‐terminal domain. In human cells, Aurora B is mainly nuclear, and it has a function to modulate cell proliferation. Aurora B is dynamically distributed during mitosis and the peak of expression level is located in the G2‐M phase.^23^ We have found through bioinformatics that Aurora B and nucleolin may have a protein interaction with each other. It was found that when the expression of nucleolin was increased, the Aurora B content decreased compared with the control group. When the expression of nucleolin decreased, Aurora B expression increased significantly. We found that there was an interaction between nucleolin and Aurora B through the protein immunoprecipitation experiments. Therefore, we hypothesized that increased expression of nucleolin would induce the binding of Aurora B, which further promotes cell proliferation and cell cycle changes, resulting in decreased expression of Aurora B in cells. And when the expression of nuceolin was decreased, there was an increase in the expression of Aurora B in the cells due to a decrease in the amount of binding between aurora B and the nucleolin. It is suggested that we are responsible for the regulation of cell proliferation due to the combination of Aurora B and nucleolin, so related expression changes have occurred.

In our study, it was demonstrated that the expressions of nucleolin in atherosclerosis plaque and cell lines were up‐regulated compared with that in normal. Restored expression of nucleolin inhibited the cell cycle of cells in vitro. In addition, we also found that Aurora B is a direct target of nucleolin. Therefore, the present result highlighted the importance of nucleolin as a vascular smooth cells proliferation by targeting Aurora B in atherosclerosis.

## CONFLICT OF INTEREST

The authors declare there are no conflicts of interest.

## AUTHOR CONTRIBUTIONS

Hui Sun: Conceptualization; manuscript drafting. Yuting Tang, Cheng Chen, Huan Chen and Zhengyang Luo: Initial drafting; instructive layout of figures. Pengfei Liang, Lingjin Huang, Xiaofang Lin and Ying Li: Constructive comments. Bimei Jiang and Xianzhong Xiao: Manuscript review; proofreading the manuscript; revision of the final version. All authors: Manuscript reading; final approved of manuscript.
